# Dissociating the functional roles of arcuate fasciculus subtracts in speech production

**DOI:** 10.1093/cercor/bhac224

**Published:** 2022-06-17

**Authors:** Nikki Janssen, Roy P C Kessels, Rogier B Mars, Alberto Llera, Christian F Beckmann, Ardi Roelofs

**Affiliations:** Donders Institute for Brain, Cognition and Behaviour, Centre for Cognition, Radboud University, PO Box 9104, 6500 HE Nijmegen, the Netherlands; Department of Medical Psychology, Radboud University Medical Center, PO Box 9101, 6500 HB, Nijmegen, the Netherlands; Donders Institute for Brain, Cognition and Behaviour, Centre for Cognition, Radboud University, PO Box 9104, 6500 HE Nijmegen, the Netherlands; Department of Medical Psychology, Radboud University Medical Center, PO Box 9101, 6500 HB, Nijmegen, the Netherlands; Vincent van Gogh Institute for Psychiatry, Centre of Excellence for Korsakoff and Alcohol-Related Cognitive Disorders, D'n Herk 90, 5803 DN, Venray, the Netherlands; Donders Institute for Brain, Cognition and Behaviour, Centre for Cognition, Radboud University, PO Box 9104, 6500 HE Nijmegen, the Netherlands; Wellcome Centre for Integrative Neuroimaging, Oxford Centre for Functional MRI of the Brain (FMRIB), Nuffield Department of Clinical Neurosciences, John Radcliffe Hospital, University of Oxford, Oxford OX39DU, United Kingdom; Donders Institute for Brain, Cognition and Behaviour, Centre for Cognition, Radboud University, PO Box 9104, 6500 HE Nijmegen, the Netherlands; Department of Cognitive Neuroscience, Radboud University Medical Centre Nijmegen, Postbus 9101, Nijmegen, 6500 HB, the Netherlands; Donders Institute for Brain, Cognition and Behaviour, Centre for Cognition, Radboud University, PO Box 9104, 6500 HE Nijmegen, the Netherlands; Department of Cognitive Neuroscience, Radboud University Medical Centre Nijmegen, Postbus 9101, Nijmegen, 6500 HB, the Netherlands; Wellcome Centre for Integrative Neuroimaging, Oxford Centre for Functional MRI of the Brain (FMRIB), Nuffield Department of Clinical Neurosciences, John Radcliffe Hospital, University of Oxford, Oxford OX39DU, United Kingdom; Donders Institute for Brain, Cognition and Behaviour, Centre for Cognition, Radboud University, PO Box 9104, 6500 HE Nijmegen, the Netherlands

**Keywords:** DTI, fMRI, language networks, arcuate fascicle

## Abstract

Recent tractography and microdissection studies have shown that the left arcuate fasciculus (AF)—a fiber tract thought to be crucial for speech production—consists of a minimum of 2 subtracts directly connecting the temporal and frontal cortex. These subtracts link the posterior superior temporal gyrus (STG) and middle temporal gyrus (MTG) to the inferior frontal gyrus. Although they have been hypothesized to mediate different functions in speech production, direct evidence for this hypothesis is lacking. To functionally segregate the 2 AF segments, we combined functional magnetic resonance imaging with diffusion-weighted imaging and probabilistic tractography using 2 prototypical speech production tasks, namely spoken pseudoword repetition (tapping sublexical phonological mapping) and verb generation (tapping lexical-semantic mapping). We observed that the repetition of spoken pseudowords is mediated by the subtract of STG, while generating an appropriate verb to a spoken noun is mediated by the subtract of MTG. Our findings provide strong evidence for a functional dissociation between the AF subtracts, namely a sublexical phonological mapping by the STG subtract and a lexical-semantic mapping by the MTG subtract. Our results contribute to the unraveling of a century-old controversy concerning the functional role in speech production of a major fiber tract involved in language.

## Introduction

For almost 150 years, disagreement has existed about the function of the fiber tracts connecting Wernicke’s area in the temporal cortex and Broca’s area in the frontal cortex. Wernicke (1874) assumed that a ventral tract connects auditory images in temporal cortex with motor images in frontal cortex via the insula (i.e. a tract passing through the extreme capsule, today known as the ventral stream of language), which he took to underpin the repetition of speech. About a century later, Geschwind (1972) assumed that a more dorsal pathway directly connecting temporal and frontal cortex, the arcuate fasciculus (AF), not only underpins speech repetition but also conceptually driven speech production, such as involved in object naming. Geschwind’s perspective has been revived by Glasser and Rilling (2008), who reported that the AF consists of 2 subtracts that appear to mediate distinct sublexical phonological and lexical-semantic processes. A computational implementation of this view has been provided by Roelofs (2014).

Using deterministic tractography, Glasser and Rilling (2008) obtained evidence that one subtract of the left AF directly connects the posterior superior temporal gyrus (STG) with the left inferior frontal gyrus (IFG), and another subtract directly connects the posterior middle temporal gyrus (MTG) and the IFG. A comparison with activations from prior functional neuroimaging (fMRI—functional magnetic resonance imaging) studies revealed the STG terminations overlapped with phonological activations and the MTG terminations with lexical-semantic activations. Based on these results, Glasser and Rilling argued that the AF is responsible for mapping both phonological representations derived from speech input (in the STG) as well as lexical-semantic representations (in the MTG) onto speech output (in the IFG) in repetition and conceptually driven language production, respectively. Recent tractography and postmortem microdissection studies have confirmed these structurally distinct STG and MTG subtracts (Fernández-Miranda et al. 2015; Yagmurlu et al. 2016). However, the hypothesized functional distinction has remained speculative because none of the previous phonological and lexical-semantic activations in the prior studies were obtained using tasks involving speech production. Also, fMRI and tractography data were acquired from different cohorts in Glasser and Rilling’s study (2008), making their conclusions about the function of the subtracts post-hoc and indirect. Hence, some authors do not assign a role in language to the direct AF connection between temporal and frontal cortex, but instead assume that 2 indirect connections via the parietal cortex mediate the repetition of speech (Ueno et al. 2011).

Here, we directly investigate the functional roles of the STG and MTG subtracts in speech production using fMRI activations and probabilistic tractography in the same cohort of participants. To functionally segregate the 2 AF subtracts, 2 different language tasks were performed. Overt repetition of aurally presented pseudowords (PR) was used to activate areas involved in sublexical phonological processes and hypothesized to be subserved by the STG subtract of the AF. In contrast, overt generation of verbs in response to aurally presented nouns (VG) was used to activate areas associated with lexical-semantic processes that are hypothesized to be subserved by the MTG subtract of the AF (Indefrey and Levelt 2004; Allendorfer et al. 2012). Task-based peak activations then served as seed regions for probabilistic tractography (Behrens et al. 2007), which enabled us to determine the most probable anatomical pathways linking activated nodes in the temporal and frontal cortex. We analyzed the inter-individual differences of fractional anisotropy (FA—a measure of tract integrity) within these 2 subtracts related to reaction time for the 2 tasks to further corroborate the differential functional contributions of these subtracts.

## Materials and methods

### Participants

Fifty cognitively unimpaired adults (25 woman, mean age 45.2 years, range 19–75, all right-handed) voluntarily participated in the experiment for monetary compensation or for course credits. All participants were native speakers of Dutch. All had normal or corrected-to-normal vision, and none had a history of central nervous system disease or language deficits (self-report). All individuals were scanned with the approval of the local ethics committee (CMO Arnhem-Nijmegen) under the general ethics approval (“Imaging Human Cognition,” CMO 2014/288), and gave their written consent.

### Behavioral tasks and materials

In the pseudoword repetition task, stimuli consisted of 100 meaningless pseudowords (e.g. *tokber* and *lart*), composed of either 1 or 2 syllables. These pseudowords were derived from Dutch words by substituting the first phoneme of each syllable on the basis of Dutch phonotactical rules, which resulted in stimuli without any meaning but with a phonemic structure typical for the Dutch language. In the verb generation task, stimuli consisted of 100 high-frequency concrete Dutch nouns (e.g. *hond* [dog] and *pistool* [gun]). The pseudowords and nouns were taken from a slightly larger set of materials that were pretested. The auditory recordings of the pseudowords and nouns were obtained from a native female speaker of Dutch.

Stimuli for both tasks were randomized using Mix (Van Casteren and Davis 2006) and were distributed into 10 blocks of 10 items each. The tasks alternated across blocks. The interstimulus interval randomly varied between 5 and 8 s in both tasks. The average pseudoword duration was 1,025 ms and the average noun duration was 946 ms. Stimuli were presented binaurally using Presentation (http://nbs.neurobs.com) and MR-compatible headphones.

### Procedure

In the pseudoword repetition task, participants were instructed to overtly repeat the pseudowords immediately after presentation. In the verb generation task, they were instructed to overtly generate an appropriate verb to the heard noun. Participants were asked to respond as quickly and accurately as possible.

At the start of each block, a reminder of the task for that block was presented on the screen using “*Zeg na*” (“Repeat”) for the repetition task or “*Noem werkwoord*” (“Name verb”) for the generation task. Each trial began with a white fixation cross on a black background, displayed continuously during stimulus presentation. Stimulus presentation was pulse-triggered and stimuli were presented aurally. The spoken responses of the participants were recorded for later determination of accuracy and reaction time. For the verb generation task, a participant’s response was considered to be correct if the generated verb was deemed appropriate to the presented noun by the first author.

### MRI data acquisition

Functional and structural MRI data from all 50 individuals were acquired on a Siemens Prisma Fit 3T scanner at the Donders Center for Cognitive Neuroimaging using a 32-channel head coil.

#### Functional MRI

Functional data consisted of 64 *T*_2_^*^-weighted slices that were recorded by Multiband gradient-echo EPI sequence with a multiband acceleration factor of 8, interleaved slice acquisition (repetition time [TR] = 735 ms, echo time [TE] = 39 ms, and field of view (FOV) = 210 × 210 mm), giving a 2.4 × 2.4 × 2.4-mm^3^ resolution.

#### Diffusion-weighted imaging

DWI was acquired with a simultaneous-multislice diffusion-weighted echo planar imaging (EPI) sequence with the following parameters: multiband factor = 3, TR/TE = 2,282/71.2 ms, in-plane acceleration iPAT = 2, voxel size = 2 × 2 × 2 mm^3^, 9 unweighted scans, 100 diffusion-encoding gradient directions in multiple shells (*b*-values = 1,250 and 2,500 s/mm^2^), and Taq = 8 min 29 s.

#### MP2RAGE

A high-resolution T1 anatomical scan was obtained for spatial processing of the fMRI and DTI data using the MP2RAGE sequence (Marques et al. 2010) with the following parameters: 176 slices, voxel size = 1 × 1 × 1 mm^3^, TR = 6 s, TE = 2.34 ms, and Taq = 7 min 32 s.

### fMRI and DWI data analysis

#### Preprocessing

All fMRI and MP2RAGE data were processed using the FMRIB software library (FSL 5.0.10, http://www.fmrib.ox.ac.uk/fsl). Preprocessing consisted of head motion correction, brain extraction, spatial smoothing using a Gaussian kernel of FWHM of 5 mm, and high-pass temporal filtering equivalent to 60 s. Each fMRI run was subjected to ICA-based automatic removal of motion artifacts (ICA-AROMA) to identify motion related components and to remove these using linear regressions (Pruim et al. 2015).

DWI images were preprocessed to realign and correct for eddy-current (SPM12) and for artifacts from head and/or cardiac motion using robust tensor modeling (PATCH; Zwiers 2010). Tensor reconstruction using weighted least squares fit was performed via DTIFit within FDT to create DTI scalar images, including the FA, MD, and 3 eigenvalues (FSL 5.0.10; Behrens et al. 2003).

#### Statistical analysis

After preprocessing, statistical analyses of fMRI data were performed at the single-subject level by using the general linear model (GLM) within the FMRI Expert Analysis Tool (FEAT) Version 6.00, from FMRIB’s Software Library (FSL; Smith et al. 2004, Jenkinson et al. 2012). Since tasks were assessed during 2 consecutive fMRI scan runs, statistical analysis proceeded through 3 levels.

At the first-level, each individual run was analyzed using GLM. In both tasks, overt response onsets were manually calculated using the software package Praat (Boersma and Weenink 2018) and incorporated into the GLM to correct for speaking related movements. Timing of the effect of interest was, thus, defined for each item by combining stimulus onset and reaction time in the first and second column of the time series file, respectively. The time series representing the effect of interest for each of the 2 tasks (PR and VG) were entered as separate regressors into the GLM. Four contrast images were produced for each participant, corresponding to the main effect of each task and the differential effects between the tasks, targeting areas where PR > VG and where VG > PR. All these images were used for the second-level analysis.

To identify brain areas activated by the differential effects between the tasks (PR > VG and VG > PR), a second-level analysis was performed combining the 2 runs from each participant. This was done using a fixed-effects model for which the coefficients (β weights) obtained for each contrast in the first-level GLM analyses were the input.

After analysis at the individual level, The FMRIB nonlinear image registration tool (FNIRT) was used to register EPI functional datasets into standard Montreal Neurological Institute (MNI)-152 space using the participant’s individual high-resolution anatomical images (MP2RAGE).

At the third-level, mixed-effects group analyses were performed for each contrast using FSL’s FLAME (FMRIB’s local analysis of mixed effects) module using the coefficients from each individual determined in the second-level GLM. The resulting statistical images were thresholded using Gaussian random field-based cluster inference with a height threshold of *Z* > 2.3 and a cluster significance threshold of *P* < 0.05.

#### Definition of seed regions

The temporal seed regions for the probabilistic fiber tracking were extracted from the *z*-statistic maps of the differential-effect contrasts of the fMRI random-effects analyses. The frontal seed regions for the probabilistic fiber tracking were defined on the *z*-statistic map based on the conjunction of the PR and VG activations. Within the major temporal and frontal activation clusters of these *z*-statistic maps the peak voxels were identified using FSL’s featquery function. Despite rigorous motion correction, these voxels of peak activation had to be defined on a group average level due to the noise at the individual level of the *z*-statistic maps. These peak voxels were then resliced to each individual’s native diffusion space by use of FNIRT, and enlarged to a sphere with a 6-mm radius. Whenever a sphere contained voxels from outside the temporal lobe, for example by spreading over the Sylvian fissure to the parietal lobe, those voxels were removed to prevent misleading connections.

### Probabilistic DTI-based fiber tracking

Fiber tracking was performed in native DT MRI space using FSL’s probtrackX (Behrens et al. 2007). After preprocessing, diffusion parameters were estimated at each voxel using BedpostX. Probabilistic tractography (ProbtrackX) was then used to estimate the distribution of connections between seed and target regions. Tracking was initiated from all voxels within the seed masks to generate 10,000 streamline samples, with a curvature threshold of 0.2 and a 0.5-mm step length. Segmentations of cerebrospinal fluid (CSF) in temporal and frontal regions, acquired through FSL FAST, were used as exclusion masks to prevent anatomically impossible tracking. Also, an extreme capsule (EmC) exclusion mask was used to prevent tracking of the ventral language pathway (Ueno et al. 2011). This resulted in probabilistic connectivity maps in which each voxel value is the total number of streamlines crossing that voxel. These connectivity distribution values were log-transformed, and normalized by dividing by the maximum tracts identified for each participant. This resulted in images with normalized connection strength (connection probability distribution maps) between 0 and 1 in each voxel and allowed us to correct for possible differences between tracts due to different dimensions of the starting seeds and to exclude the background noise. The normalized connection maps were thresholded at 0.5 (following earlier studies; Mars et al. 2016) to exclude spurious connections. The connectivity maps were then warped to MNI space and an average connectivity map was created.

**Fig. 1:**
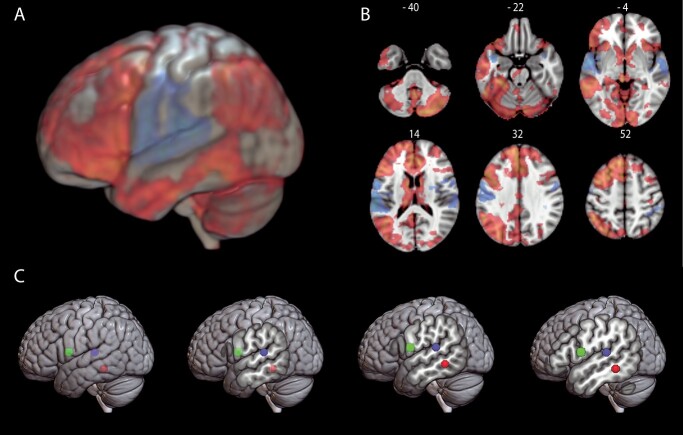
fMRI results. Functional networks involved in pseudoword repetition > verb generation (blue–light blue) and verb generation > pseudoword repetition (red–yellow). Statistical activation maps displayed A) on an MNI152 template brain in 3D and B) in a selection of axial slices with corresponding slice numbers. C) Spheres were created based on peak voxels within the temporal and frontal clusters and served as seed regions for the probabilistic fiber tracking. Seed based on verb generation in red, seed based on pseudoword repetition in blue, and seed based on frontal activation in both tasks in green.

### Validation of subtracts

To assess the resulting connectivity maps in a quantitative way, we subsequently focused on the region where the fMRI-based tracts arc around the lateral sulcus and calculated the locality of peak sample count of the group probability maps of each tract using Fslstats. For validation purposes, we then performed linear discriminant analyses (LDA) using as features the *xyz*-coordinates of the peak voxel count within the arc of the AF for each task and each subject and assessed the performance using leave one out (LOO) cross-validation.

### Linking structure to function

A paired sample *t*-test was used to assess functional differences between tasks by testing for reaction time and error rate differences. The relationship between structure and function of the AF subtracts was assessed by using a GLM multivariate regression within the FEAT Version 6.00, from FMRIB’s Software Library (FSL; Behrens et al. 2003; Smith et al. 2004). The MNI-aligned FA image of each participant were merged into a 4D FA data set and fed into this GLM-model, in which the time series representing the reaction time for each of the 2 tasks (PR and VG) were entered as separate independent explanatory variables. Age was included as a regressor of no interest to correct for age effects on both FA as well as task RT. This GLM produced 2 contrast images, corresponding to the differential effects between the tasks (PR > VG and VG > PR) related to reaction time, targeting areas where FA was related to reaction time per task. The resulting *z*-statistic images were cropped by the masks of the 2 arcuate subtracts and a 2-sample *t*-test was used with the null hypothesis that the *z*-statistic images within each subtract mask have equal means, against the alternative that the means are not equal.

## Results

### fMRI activations

The comparison of pseudoword repetition versus verb generation (PR > VG) revealed strong bilateral (left more than right) temporal and frontal activations with peak activations in the STG (including the planum temporale) and primary motor areas (Fig. 1A and B). This effect defined the temporal areas underpinning the sublexical phonological mapping. Strongest activation was observed in the posterior STG (*xyz*-coordinates: −62, −30, and 14).

When contrasting verb generation with pseudoword repetition (VG > PR), a more widespread pattern of activation was seen, with peak activations observed in the left MTG, the anterior cingulate gyrus, and left frontal areas including the IFG (Fig. 1A and B). This effect defined the areas underpinning the lexical-semantic mapping. The temporal peak with the highest activation strength was located in the MTG (*xyz*-coordinates: −60, −42, and −10). These STG and MTG activations for repetition and generation, respectively, correspond to the phonological and lexical-semantic activations that Glasser and Rilling (2008) derived from prior fMRI studies in the literature, albeit from studies not involving speech production.

The pars opercularis of the IFG is assumed to be common to both subtracts and related phonological and lexical-semantic mappings (Glasser and Rilling 2008). Therefore, rather than using the contrasts between repetition and generation (which would give disjoint activations), we obtained the peak with the highest activation strength in the IFG common to both tasks, which was in the IFG pars opercularis (*xyz*-coordinates: −58, 4, and 12).

**Fig. 2:**
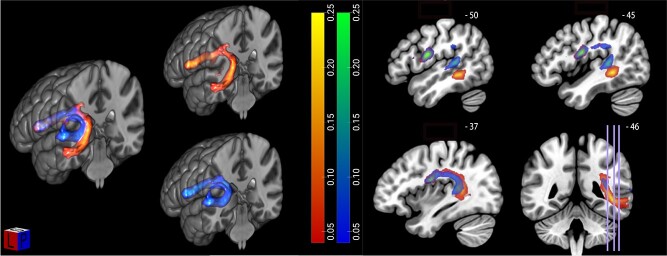
Tractography results visualized in MRIcroGL. Left: Composite fiber networks subserving repetition (in blue–light blue) and verb generation (in red–yellow) in the left hemisphere computed by averaging the normalized and thresholded (0.5) connection maps of 50 subjects based on seeds defined in the pseudoword repetition and verb generation tasks. Scale = 0–0.25; opacity = 50%. Right: Corresponding sagital and coronal slices.

### fMRI-guided tractography

From the fMRI-based peak voxels of activation, we created 2 ROIs per task, one temporal and one frontal, to initiate fiber tracking in the native diffusion space of every participant (Fig. 1C). To identify fiber pathways linking the activated nodes for the sublexical phonological mapping during pseudoword repetition, probabilistic connectivity maps were generated from the posterior STG seed to the IFG pars opercularis seed. In the same way, probability maps were generated from the posterior MTG seed to the IFG pars opercularis seed to identify fiber pathways linking the activated nodes for the lexical-semantic mapping during verb generation.

The participant-specific tracts for each task, acquired from the probabilistic connectivity maps, were warped to MNI space and an average connectivity map was created (Fig. 2). In the left panel of Fig. 2, both the tracts connecting the fMRI-based seeds in the left posterior STG and IFG pars opercularis for the pseudoword repetition task as well as the tracts connecting the fMRI-based seeds in the left posterior MTG and IFG pars opercularis for the verb generation task are shown. For both tasks, seeds are connected via the AF, and when these tracts are shown in combination, a clear dissociation of the AF into 2 subtracts becomes evident (Fig. 2). The subtract connecting the pseudoword repetition (PR > VG) seeds forms a fiber pathway running shallowly under the cortex, whereas the subtract connecting the verb generation (VG > PR) seeds courses more posterior and medially to it. The spatial course of these subtracts corresponds to the findings of earlier tractography and postmortem microdissection studies in the literature (Glasser and Rilling 2008; Fernández-Miranda et al. 2015; Yagmurlu et al. 2016).

**Fig. 3:**
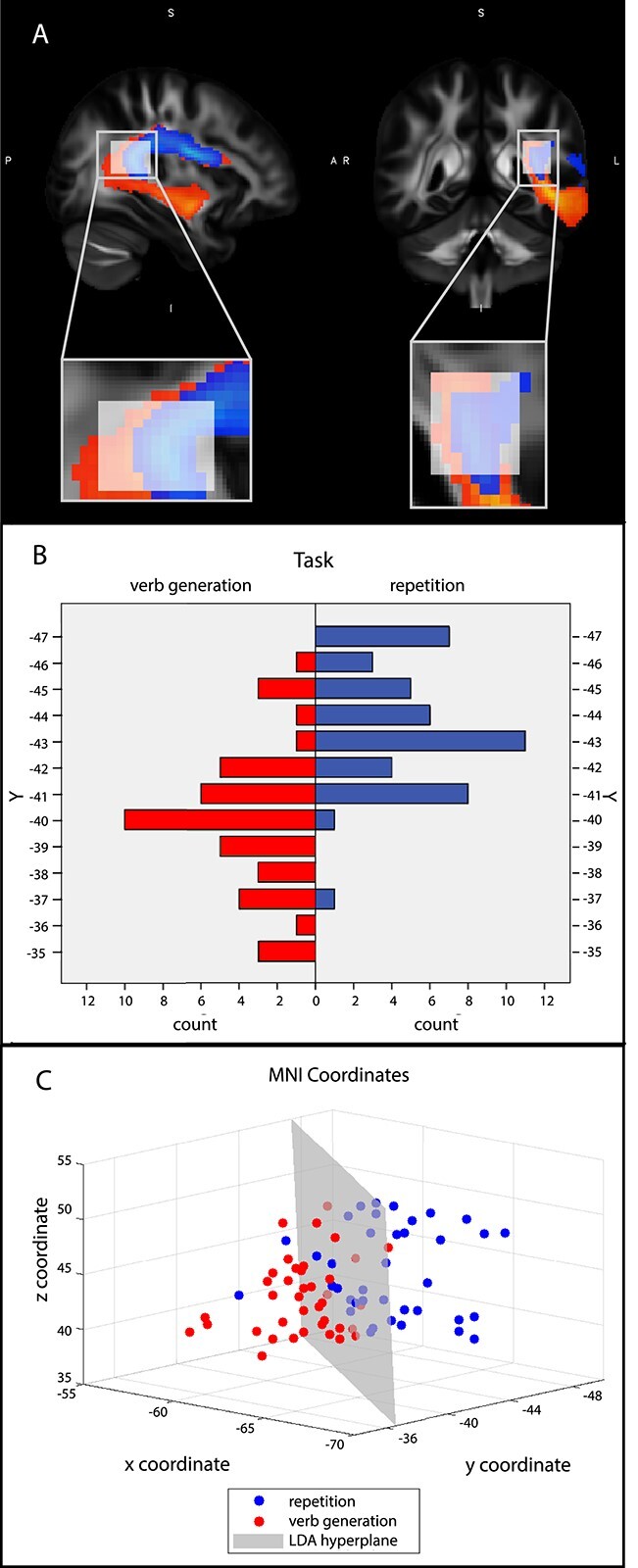
A) Region of interest for LDA, where the fMRI-based AF subtracts arc around the lateral sulcus. B) Histograms of anterior–posterior distributions (*y*-coordinates) of the peak voxel count within the arc of the AF for each task for all subjects. C) Dots represent the 3D coordinates of the peak count voxels per subject and per task; blue dots represent pseudoword repetition and red dots verb generation. In gray a decision plane learned by the 3-dimensional LDA is shown.

### Validation of the AF-subtracts

Since the fMRI-based tractography revealed a clear segregation within the left AF of 2 subtracts (Fig. 2), further steps to validate this observation were undertaken. First, the location of the peak sample count of the group probability maps of each tract was calculated within the region where the fMRI-based tracts arc around the lateral sulcus (Fig. 3A) as this region showed the most anatomically significant cross-subject variation. The voxels of peak sample count of each of these subtracts showed a clear difference in distribution of *y*-coordinates within the arc of the AF (Fig. 3B).

Subsequently, this segregation into subtracts was confirmed by a LDA using the *xyz*-coordinates of the peak sample count within the arc of the AF for each task and each participant as features. The LDA resulted in a mean classification accuracy of 82.9% (STD = 0.29) under cross-validation, demonstrating that the location of peak sample count within the AF contains discriminative power to distinguish which of both tasks was being performed (Fig. 3C). The *x*, *y*, and *z* coordinates received LDA weights equal to 0.53, 0.70, and 0.02 respectively, indicating that the *z*-axis is not informative for discrimination between peak locations. Indeed, using only the *x* and *y* coordinates resulted in a slight increase in performance, mean classification accuracy of 84.15% (STD = 0.26).

### Linking AF-structure to function

A paired sample *t*-test showed reaction times to be significantly longer for the verb generation task (*M* = 1.82 s, *SE* = 0.3) than for the pseudoword repetition task (*M* = 1.57 s, *SE* = 0.25), *t*(49) = 11.54, *P* < 0.001, *r* = 0.85. There was no statistical difference in number of errors between tasks (*P* > 0.05). A multiple linear regression analysis of FA against reaction time, including age as regressor of no interest, for the 2 different tasks was applied to further establish the differential functional contributions of the MTG and STG subtracts. The 2-sample *t*-test comparing the mean *z*-statistic within the 2 AF subtract masks for each of the 2 contrasts (VG > PR and PR > VG related to reaction time) showed significant dissociations between the subtracts and the different tasks. The inter-individual differences in FA within the MTG (verb generation) subtract are significantly better explained by the reaction time for VG than for PR (*t* = 11.0, *P* < 0.001). Conversely, the inter-individual differences in FA within the STG (repetition) subtract are significantly better explained by the reaction time for PR than for VG (*t* = 11.65, *P* < 0.001).

### Specificity of our findings for the AF

The results obtained for the connectivity of the AF subtracts with the pars opercularis were replicated for the pars triangularis (see Supplementary analysis 1), indicating shared functionality. Moreover, additional analysis showed that the RT findings on VG versus PR better explain the inter-individual differences in FA within AF subtracts than within the superior longitudinal fasciculus (SLF) subtracts II and III with parietal seeds (Supplementary analysis 2), demonstrating the specificity of our findings for the AF.

**Fig. 4:**
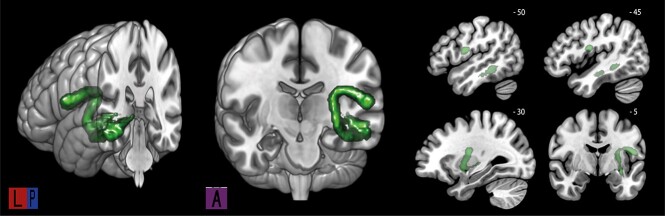
Tractography results for the EmC fiber pathway averaged over all 50 participants.

Finally, to examine the role of the extreme capsule (EmC) pathway, also known as the ventral language pathway, we performed an additional analysis using the same data-driven temporal (STG/MTG) and frontal seeds as in our analyses on the AF tracts. However, in this new analysis, the AF was used as an exclusion mask while the EmC exclusion was removed. In this way, the seeds are still based on the fMRI task activations, but forced to focus on the EmC connections only. As this pathway has been suggested to play a role in top-down control of lexical-semantic processing in speech production (e.g. Roelofs 2014; Janssen et al. 2020), especially the relation of the EmC fiber connections from MTG to frontal seeds are of interest. These connections are visualized in Fig. 4. It should be noted that this tract does not look like the traditional ventral pathway, possibly because the tractography is artificially being forced not to use the dorsal route.

The analysis showed that, just as for the MTG subtract of the AF, the FA values of the MTG tract of the EmC are better explained by the reaction time for VG than for PR compared with the STG subtract of the AF (*t* = 9.4, *P* < 0.001). In addition, there is no significant difference in the relation between the inter-individual differences in FA within the MTG tract of the EmC and the MTG subtract of the AF with regard to the VG reaction time (*t* = 0.83, *P* = 0.41). These results indicate that the MTG subtract of the AF and the MTG tract of the EmC both play a role in verb generation, indicating a role for the EmC pathway in lexical-semantic processing in speech production.

## Discussion

To date, no study has directly investigated the functional role of the 2 AF subtracts in speech production. To assess this relationship between AF structure and function, we combined diffusion-weighted MRI and probabilistic tractography with fMRI to extract the most probable dorsal white-matter pathways connecting functionally specified speech production areas. We observed that left superior temporal and inferior frontal regions activated during pseudoword repetition are linked via the STG subtract of the AF, and additionally, that microstructural properties of this subtract are related to functional measures of the pseudoword repetition task. In contrast, left middle temporal and inferior frontal regions activated during conceptually driven verb generation are linked via the MTG subtract of the AF, and microstructural properties of this subtract are related to functional measures of the verb generation task.

Our findings support the functional account of the STG and MTG subtracts of the AF by Glasser and Rilling (2008), who proposed that these subtracts mediate sublexical phonological and lexical-semantic mappings, respectively. However, the evidence advanced by Glasser and Rilling remained indirect, because their functional activations were derived from prior studies that involved different groups of participants and did not involve speech production.

To accommodate these issues, tractography in the present study was based on cortical activations obtained in a single group of participants through task-based fMRI using 2 overt speech production tasks specifically engaging the sublexical phonological and lexical-semantic mappings, namely pseudoword repetition and verb generation. Furthermore, by employing a probabilistic tracking algorithm we can account for the possibility of crossing fibers and subject movement (Behrens et al. 2007).

In the pseudoword repetition task, activation focused on left STG, premotor, and prefrontal areas, replicating the findings on speech repetition obtained by Saur et al. (2008). In the verb generation task, a more widespread pattern of activations was found, as can be expected for conceptually-driven word production (e.g. Indefrey and Levelt 2004). This difference in functional processing demands was also reflected behaviorally, as reaction times were significantly longer for the verb generation task than for the pseudoword repetition task. Besides the extent of activated areas, each task also showed different locations of peak activation within the temporal lobe, namely posterior STG for pseudoword repetition and posterior MTG for verb generation, in agreement with Glasser and Rilling’s (2008) functional activations derived from prior studies.

Catani et al. (2005) proposed a DTI-based classification for the AF, separating the fascicle into a long segment running directly from temporal to frontal cortex and 2 indirect segments via parietal cortex, namely a posterior temporo-parietal segment and an anterior parietal–frontal segment. According to Catani and colleagues, the long segment underpins speech repetition and the indirect segments underpin conceptually driven naming. In contrast, Glasser and Rilling (2008) and Roelofs (2014) argue that the AF segment that directly connects temporal to frontal areas underlies both speech repetition as well as conceptually driven speech production. The function of the parietal cortex and the indirect temporo-parietal and parietal–frontal connections of the AF in speech production remains a topic of debate. The current study focused on the direct segment of the AF connecting temporal and frontal cortex, and does not include functional assessment of the indirect temporo-parietal and parietal–frontal connections of the AF. The evidence for the existence of 2 functionally distinct direct temporo-frontal subsegments of the AF obtained in the present study supports the view by Glasser and Rilling (2008) and Roelofs (2014) and calls into question the view that the long AF segment is solely involved in speech repetition, as suggested by Catani et al. (2005).

In discussing the AF, Ueno et al. (2011) only considered the 2 indirect parietal segments, which they took to mediate primarily the repetition of speech (different from Catani et al. 2005). Evidence from aphasia patients reported by Kümmerer et al. (2013), Marchina et al. (2011), and Wang et al. (2013) and the tractography evidence obtained by Saur et al. (2008) and in the present study indicates that speech repetition is instead, or in addition, mediated by a direct connection between the temporal and frontal cortex.

In addition, there is a debate about whether lexical-semantic information from temporal language areas (including MTG) is conveyed to the IFG via a ventral or dorsal pathway (Ueno et al. 2011; Roelofs 2014; Fridriksson et al. 2018). The aim of the present study was to assess the functional involvement of the dorsal AF pathway in conceptually driven speech production. Our results indicate that the MTG subtract is involved in the lexical-semantic mapping during speech production. However, as the AF shows asymmetry and subject to subject variability (Catani et al. 2007), our results do not rule out alternative routes of structural connectivity implicated in speech production. To examine the role of the EmC pathway, also known as the ventral language pathway (Ueno et al. 2011), we performed an additional analysis using the same data-driven temporal and frontal seeds as in our analyses on the AF tracts. We observed that an EmC tract running from MTG to frontal gyrus is implicated in VG, suggesting a role for the EmC pathway in lexical-semantic processing. Whereas Ueno et al. (2011) maintained that the EmC pathway conveys lexical-semantic information to the IFG in speech production, Roelofs (2014) argued for a role of the EmC in top-down control of lexical-semantic processing (for recent empirical evidence, see Janssen et al. 2020). The current findings do not adjudicate between these 2 views. Although the current study clearly points towards an important role for the dorsal (AF) pathway in speech production, the complex process of speech production likely unfolds via complex interactions within a larger network comprising both gray- and white-matter structures (Axer et al. 2013). In naturally occurring speech, both ventral and dorsal networks probably interact closely to reach high competence in verbal communication (e.g. Roelofs 2014; Janssen et al. 2020). Future studies are needed to unravel this complex network and the interplay of its components in more detail.

Our observation that the AF mediates both pseudoword repetition and verb generation is in line with the classic view of Geschwind (1972) that the AF underlies both speech repetition and conceptually driven naming. This view is supported by the evidence that damage to the AF may not only impair speech repetition but also object naming (Marchina et al. 2011; Wang et al. 2013; Hope et al. 2016; Geller et al. 2019). However, Geschwind did not assume distinct subtracts of the AF for these 2 language tasks as we observed. As argued by Glasser and Rilling (2008) and demonstrated by computer simulations (Roelofs 2014), the assumption of spatially separate AF subtracts may explain double dissociations between repetition of speech and conceptually driven production of speech observed in aphasia. These dissociations are more difficult to accommodate by the view on the AF of Geschwind.

In summary, our findings provide direct evidence for distinct functional roles in speech production of subtracts within the left AF. These subtracts link the posterior STG and MTG to the posterior IFG. We observed that the repetition of spoken pseudowords is mediated by the STG subtract and that the generation of a verb that is appropriate to a spoken noun is mediated by the MTG subtract. These findings suggest that a sublexical phonological mapping is underpinned by the STG subtract and a lexical-semantic mapping by the MTG subtract, supporting the dual-stream view on the AF of Glasser and Rilling (2008) and Roelofs (2014).

## Supplementary Material

Supplementary_data_bhac224
