# Supplementary material for: Dissociating the functional roles of arcuate fasciculus subtracts in speech production
Source: Cereb Cortex. 2022 Jun 17;33(6):2539–47. doi: 10.1093/cercor/bhac224 (PMC10016035; doi:10.1093/cercor/bhac224)
Supplement: Supplementary_data_bhac224 [file supplementary_data_bhac224.docx]

**Supplementary Data**

**Dissociating the Functional Roles of Arcuate Fasciculus Subtracts in Speech Production**

**Janssen, N., Kessels, R. P. C., Mars, R. B., Llera, A., Beckmann, C. F., & Roelofs, A.**

**Supplementary Analysis 1: Arcuate Fasciculus (AF) Subtract Connectivity with Pars Triangularis**

In the article, seeds for tractography were defined in a data-driven way as they were based on the location of peak fMRI activation during both the verb generation (VG) and pseudoword repetition (PR) tasks. For the frontal seed, this location was found to be within the pars opercularis and not the pars triangularis. Here, we report whether the arcuate fasciculus (AF) subtracts from superior temporal gyrus (STG) and middle temporal gyrus (MTG) also connect to the pars triangularis and relate to the behavioral data.

In a new analysis, the frontal seed was based on the location of peak fMRI activation for both the VG and PR tasks within the pars triangularis only. The tractography results are visualized in Supplementary Figure 1.


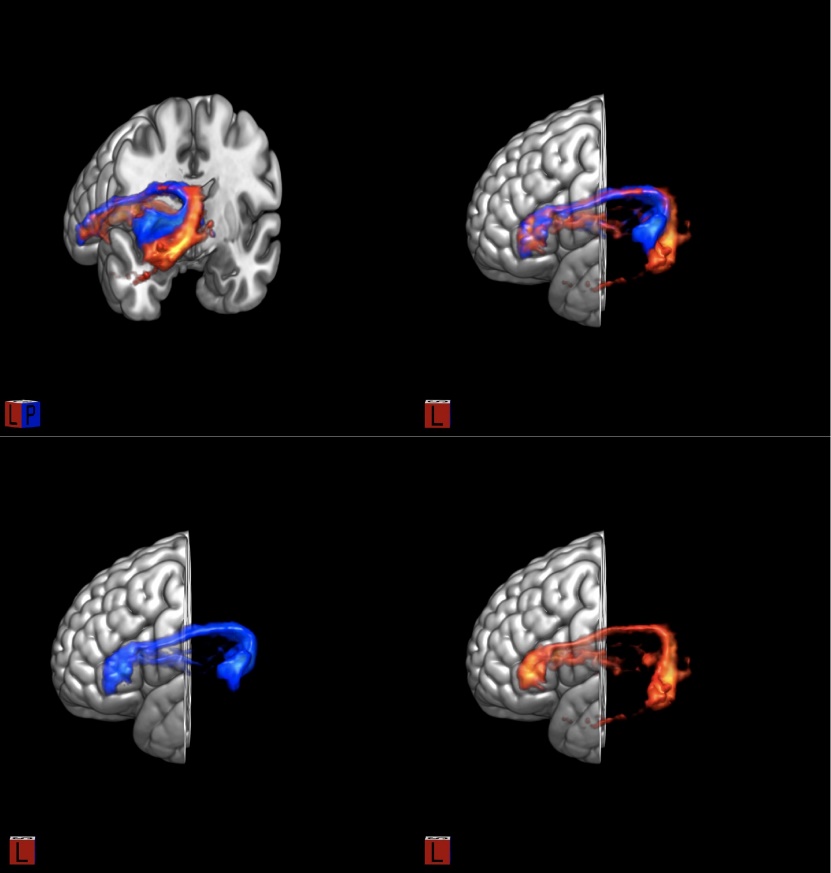


**Supplementary Figure 1**. Composite fiber networks consisting of an STG tract in blue-light blue (repetition) and an MTG tract in red-yellow (verb generation) in the left hemisphere computed by averaging connections of 50 participants with the peak-sphere in the frontal pars triangularis.

We found a link between structure and function for these subtracts connecting to the pars triangularis, as the inter-individual differences in FA within this new MTG (verb generation) subtract were significantly better explained by the reaction time for VG than for PR (*t* = 4.4, *p* < .001). Conversely, the inter-individual differences in FA within the new STG (repetition) subtract were significantly better explained by the reaction time for PR than for VG (*t* = 2.5, *p* = .01).

To conclude, the results obtained for the connectivity of the AF subtracts with the pars opercularis are replicated for the pars triangularis.

**Supplementary Analysis 2: Role of Superior Longitudinal Fasciculus (SLF) Subtracts II and III**

To prove specificity of our findings for the AF, we performed additional analyses tracking the Superior Longitudinal Fasciculus (SLF) subtracts II and III as control tracts and the Inferior Parietal Lobe (IPL) as seed region by use of FSL’s Xtract (Warrington et al., 2020). The tractography results are visualized in Supplementary Figures 2 and 3.


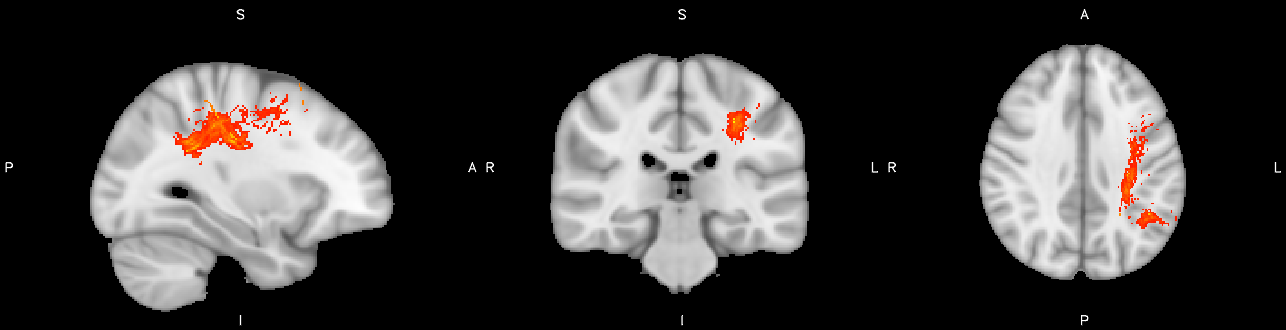


**Supplementary Figure 2**. Tractography results for the SLF II. Shown is the average over all 50 participants, thresholded at P50 for visualization purposes.


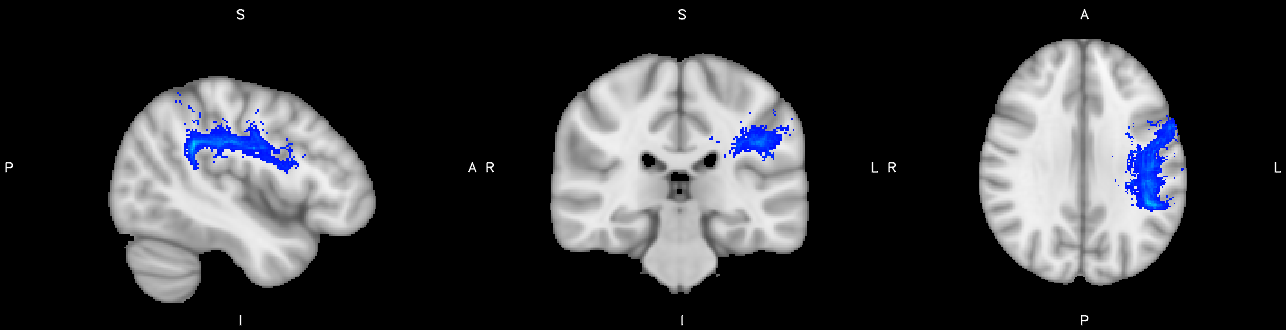


**Supplementary Figure 3**. Tractography results for the SLF III. Shown is the average over all 50 participants, thresholded at P50 for visualization purposes.

Subsequently, we investigated the link between structure and function for the SLF II and SLF III compared to the AF subtracts connecting to the pars opercularis. We found that FA values within the AF subtracts are significantly better explained by the reaction times of the VG and PR tasks than the FA values of the SLF II and SLF III. In particular, the inter-individual differences in FA within the MTG subtract of the AF were significantly better explained by the reaction time for VG than for PR compared to the SLF II (*t* = −25.9, *p* < .001) and the SLF III (*t* = −24.5, *p* < .001). Conversely, the inter-individual differences in FA within the STG subtract of the AF were significantly better explained by the reaction time for PR than for VG compared to the SLF II (*t* = −9.3, *p* < .001) and the SLF III (*t* = −11.9, *p* < .001).

These results show the specificity of the findings on the AF subtracts, and suggest the subtracts underlying verb generation and repetition to be part of the AF and not the SLF.

**References**

Janssen N, Roelofs A, Mangnus M, Sierpowska J, Kessels RPC, Piai V. 2020. How the speed of word finding depends on ventral tract integrity in primary progressive aphasia. *NeuroImage Clinical*. 28:102450.

Roelofs A. 2014. A dorsal-pathway account of aphasic language production: The WEAVER++/ARC model. *Cortex*. 59:33–48.

Warrington S, Bryant KL, Khrapitchev AA, Sallet J, Charquero-Ballester M, Douaud G, Jbabdi S, Mars RB, Sotiropoulos, SN. 2020. XTRACT - Standardised protocols for automated tractography in the human and macaque brain. *NeuroImage*. 217:116923.
